# Contrasting microbial community responses to salinization and straw amendment in a semiarid bare soil and its wheat rhizosphere

**DOI:** 10.1038/s41598-019-46070-6

**Published:** 2019-07-05

**Authors:** Márton Szoboszlay, Astrid Näther, Bei Liu, Angel Carrillo, Thelma Castellanos, Kornelia Smalla, Zhongjun Jia, Christoph C. Tebbe

**Affiliations:** 1 Thünen Institute of Biodiversity, Braunschweig, Germany; 2 Institute of Soil Science, Chinese Academy of Science, Nanjing, Jiangsu China; 3 Centro de Investigaciónes Biologicas del Noroeste (CIBNOR), La Paz, Baja California Sur, Mexico; 4 Julius Kühn Institute of Epidemiology and Pathogen Diagnostics, Braunschweig, Germany

**Keywords:** Soil microbiology, Agroecology

## Abstract

Soil salinization is a major constraint of agriculture in semiarid ecosystems. In this study soil microcosms were applied to evaluate the impact of a lower- and higher-level salinization treatment of a pristine scrubland soil on the abundance of *Bacteria*, *Archaea*, and *Fungi*, and on prokaryotic diversity in bare soil and the rhizosphere of wheat assessed by qPCR and high-throughput sequencing of 16S rRNA gene amplicons. Furthermore, the impact of soil straw amendment as a salt-stress alleviation strategy was studied. While the low-level salinity stimulated plant growth, the seedlings did not survive under the higher-level salinity unless the soil was amended with straw. Without the straw amendment, salinization had only minor effects on the microbial community in bare soil. On the other hand, it decreased prokaryotic diversity in the rhizosphere of wheat, but the straw amendment was effective in mitigating this effect. The straw however, was not a significant nutrient source for the rhizosphere microbiota but more likely acted indirectly by ameliorating the salinity stress on the plant. Members of *Proteobacteria*, *Actinobacteria*, and *Firmicutes* were abundant among the bacteria that reacted to soil salinization and the straw amendment but showed inconsistent responses indicating the large physiological diversity within these phyla.

## Introduction

In semiarid ecosystems, the extension of agricultural land area for cultivation of crops has caused the replacement of the original vegetation, typically scrublands, and thereby a dramatic loss of floral and faunal biodiversity^1,2^. Converting scrublands to croplands strongly effects soil properties introducing frequent irrigation and mixing by tillage to previously water-limited, stratified systems with low nutrient input^3,4^. Under agricultural management, the land area also receives fertilizers, pesticides, and organic matter from crop residues. It has been shown that conversion from scrubland to cropland causes a loss of soil organic carbon and phosphate but an increase in soil salinity^5^. While the lost phosphate can be substituted by fertilization, the loss of carbon and the increasing salinity can impede agricultural productivity, especially by low quality irrigation water and poorly drained soils^6^.

Soil salinization is one of the major constraints for agriculture in semiarid ecosystems and threatens food security on a global scale with affected areas spreading annually and predicted to reach 50% of arable land by 2050^7,8^. Economically important crops show significant decrease in yield above a salinity threshold of 1 to 8 dS m^−1^ depending on the species, with wheat and barley being more tolerant than maize and most vegetables^9^. Plants respond to salinity stress by osmotic adjustment accumulating solutes to balance the osmotic pressure^10^. This, however, has a substantial energy demand that restricts plant growth^11^. In addition, the accumulation of sodium ions in the leaves has a direct inhibitory effect on photosynthesis^12^. There are also below-ground responses: reduced primary root growth but increased lateral root development has been found in durum wheat and *Arabidopsis thaliana* under salt stress^13,14^. When facing salinity stress, a plant may benefit from the activity of the microbial community colonizing its rhizosphere^15,16^. For instance, the bacterial production of extracellular polymeric substances (EPS) can aid the plants in resisting drought and salinity^17,18^. Beyond the direct adverse effects of salinity on plants, it also destabilizes soil structure through replacing bivalent cations like calcium (Ca^2+^) by sodium (Na^+^) on the cation exchange sites^19^. As a consequence, salinized soils do not only lose soil organic carbon but also their structural stability further decreasing soil fertility.

Strategies to maintain agricultural productivity and to alleviate the salinity stress include the development of salt-tolerant cultivars^20,21^ and the implementation of alternative cropping systems^22^ and specific management practices, e.g., drip-irrigation or soil drainage^23,24^. Salinity stress can also be reduced by amending soils with organic substances like manure, composts, or straw^25–28^. The introduction of such particulate organic materials can have several beneficial effects including improvement of soil structure and promotion of growth of microbial biomass that provides EPS and enzymatic activities which facilitate soil aggregate formation and the stabilization of organic carbon^29^.

Considering the importance of microbiologically mediated activities to support plant growth and improve soil structure in salinity affected soils, our objective in this study was to analyze how salinity affects microbial abundance and prokaryotic diversity in bare soil and in the rhizosphere of wheat in soil from a semiarid ecosystem, and how amending the soil with straw modifies these effects. More specifically, we conducted a two phase experiment under greenhouse conditions with scrubland soil from a semiarid ecosystem without any history of agricultural use which was either amended with straw or not before the experiment: During the first phase, the soil received either non-saline water, or water with low salinity or high salinity allowing evaporation but excluding drainage to cause the accumulation of salts as it is common for semiarid ecosystems^24^. Subsequently, wheat seedlings were planted into the soil and grown for 7 weeks to obtain rhizosphere samples.

We hypothesized that (1) the addition of saline water transiently increases microbial abundance in bare soil due to the mobilization of carbon but, as a selective factor, reduces prokaryotic diversity in both the bare soil and in the rhizosphere. Furthermore, we expected that (2) straw amendment provides an additional carbon and energy source resulting in increased microbial abundance and ameliorating the adverse effect of salinity on soil prokaryotic diversity. The microbial communities in the bare soil and rhizosphere samples were characterized by high-throughput sequencing of 16S rRNA gene amplicons to assess the diversity of *Bacteria* and *Archaea*, and by qPCR of 16S rRNA genes as an estimate of the abundances of *Bacteria* and *Archaea*, and ITS sequences to estimate fungal population sizes.

## Materials and Methods

### Soil

The soil was a Xerosol collected from a scrubland located in the La Paz-El Carrizal basin, 12 km west of the city of La Paz, Baja California, Mexico on November 13, 2012. The sampling site was within a protected area without any previous history of agricultural use (GPS position: 24°07′43″N, 110°26′03″W). Details about the vegetation and soil properties have been described elsewhere^30,31^. Briefly, the soil had pH 7.9, electrical conductivity (EC1:5) 0.41 dS m^−1^, total carbon 3.0 mg C g^−1^ soil dry weight (d.w.), total nitrogen (Nt) 0.7 mg N g^−1^ d.w., and water holding capacity (WHC) 27% (w/w) g^−1^ d.w. After sampling, the soil was sieved (mesh size 2 mm), air-dried, and kept for 15 weeks at room temperature before the experiment.

### Experimental setup

The experiment consisted of two phases. In the first phase a salinization treatment was performed: soil with or without straw amendment was irrigated with non-saline water or water with low or high salinity to reach three different levels of soil salinity (control, low, high). In the second phase, wheat seedlings were grown in the soil to obtain rhizosphere samples. At the start of phase 1, glass beakers (0.2 L) were filled with 100 g soil or soil amended with straw. The straw was a mixture of equal amounts of dried whole plant material from maize “Simao”, sorghum “Tarzan”, and summer wheat “Ethos” ground to a particle size of 1 mm. The material contained 48% (w/w) carbon and 0.8% (w/w) nitrogen, and was mixed to the soil to reach 2% (w/w). The salinization treatment was achieved by irrigating the soil with water with different levels of salinity: the controls received sterile distilled water (0 dS m^−1^), in the low salinity treatment sterile distilled water with 1.125 g NaCl L^−1^ (1.9 dS m^−1^) was used, while in the high salinity treatment sterile distilled water with 7 g NaCl L^−1^ (10.8 dS m^−1^) was applied. Each combination of straw amendment and salinity treatment was replicated four times resulting in 24 glass beakers with soil in total. The soil was irrigated according to the salinization treatment once a week for eight weeks maintaining 50% WHC. For an additional three weeks, all beakers were irrigated with non-saline sterile distilled water to allow the equilibration of the soil conditions. The soil was then removed from each beaker, sieved (2 mm), and divided into two parts: 60 g was filled back into the beaker while 40 g was collected and analyzed as phase 1 bare soil sample. An aliquot of 2 g from each sample was frozen and kept at −80 °C until DNA extraction. The remaining 38 g of each sample was air-dried and used for chemical analyses. In phase 2, three seedlings of the wheat cultivar Opata, germinated on wet filter paper four days before, were planted into the remaining 60 g of soil in each beaker. The plants were watered 2–3 times a week according to their needs with non-saline water and fertilized weekly with 0.06 mg g^−1^ soil d.w. Wuxal Basis Suspension (Schering AG, Düsseldorf-Heerdt, Germany). The phase 2 rhizosphere samples were collected after 7 weeks of plant growth. Plants were uprooted and loosely adhering soil was removed from the roots by shaking. Roots of the three individual plants per beaker were combined and washed in 30 ml sterile saline (0.85% NaCl) for 30 min at 4 °C in an orbital shaker (Model 3040, GFL, Burgwedel, Germany) with 10 rpm. The roots were then removed and the cells were collected from the solution by centrifugation at 4,100 × *g* for 30 min at 4 °C. Pellets were stored at −80 °C until DNA extraction. The remaining soil (phase 2 bulk soil) was sieved (2 mm) and air-dried for chemical analyses. The roots and shoots were oven-dried and weighed to measure plant biomass. The seedlings did not survive in the soil from the high salinity treatment without straw amendment. Additionally, the plants died in one beaker from the control treatment without straw amendment, hence only three replicates were sampled from this treatment combination.

### Soil chemical and physical analyses

The electrical conductivity (EC) as an indicator for salinity was measured as dS m^−1^ in a 1:5 soil-to-water ratio mixture after 1 h of shaking on an orbital shaker^32^. Soil pH was determined in 0.01 M CaCl_2_ using a soil-to-solution ratio of 1:2 (w/v). Total carbon (C_t_ [%]) and nitrogen (N_t_ [%]) were measured via dry combustion using an elemental analyzer (LECO TruMac, Elementar, Germany). The water holding capacity (WHC; % H_2_O g^−1^ w/w) was measured gravimetrically after allowing thoroughly wetted soils to drain on a sand bed for 3 h. Details of the methods can be found elsewhere^33^.

### DNA extraction

DNA was extracted using the FastDNA SPIN kit for soil (MP Biomedicals, Illkirch, France). The extraction included two bead beating steps of 45 s at 6.5 m s^−1^ on a FastPrep-24 system (MP Biomedicals) and one additional washing step of the binding matrix with 1 ml 5.5 M guanidine thiocyanate (Carl Roth, Karlsruhe, Germany). DNA extraction failed from a phase 2 rhizosphere sample from the control treatment with straw amendment; therefore, there were only three replicates from this treatment combination.

### Quantification of the bacterial, archaeal, and fungal communities

Population sizes of the bacterial and archaeal communities were determined by quantitative real-time PCR applying the Maxima Probe qPCR ROX Master Mix (Thermo Fisher Scientific, Epsom, UK) with 0.5 mM of each of the primers and 0.2 mM of the FAM-labeled TaqMan probe. Fungi were quantified with the Maxima SYBRGreen/ROX qPCR Master Mix (Thermo Fisher Scientific) and 0.5 mM of each primer. The amplification was followed by a melt curve analysis. A total of 2 µl of template DNA diluted 50-fold in TE-buffer (10 mM Tris, 1 mM EDTA, pH 8) were used in each 20 µl reaction. All reactions were performed in duplicates in a StepOnePlus Real Time PCR System (Life Technologies GmbH, Darmstadt, Germany). Standard curves were obtained from 10-fold dilutions of the pGEM-T vector (Promega, Mannheim, Germany) containing the 16S rRNA gene of *Bacillus subtilis*, or *Methanobacterium oryza*, or the ITS of *Fusarium culmorum*. PCR conditions, primer and probe sequences with references are listed in the Table S1. The average PCR efficiency was 98% for *Bacteria* and *Archaea* and 85% for *Fungi* with R^2^ > 0.99.

### Illumina PCR-amplicon sequencing and data processing

The V4 region of the 16S rRNA gene was amplified with primers S-D-Arch-0519-a-S-15 (5′-CAGCMGCCGCGGTAA-3′) and S-D-Bact-0785-a-A-21 (5′-GACTACHVGGGTATCTAATCC-3′)^34^ and sequenced according to the protocol of Kozich *et al*.^35^. Primer sequences are listed in the Table S2. Two PCR amplifications were carried out from each DNA extract with the FastStart High Fidelity PCR System (Roche Diagnostics, Mannheim, Germany) in 50 µl final volume. Each reaction contained 1 µl template DNA, 0.4 µM of each primer, 200 µM of each dNTP, 5% dimethyl sulfoxide and 2.5 U FastStart High Fidelity Enzyme Blend in a reaction buffer with 1.8 mM MgCl_2_. The temperature program of the reactions was initial denaturation at 95 °C for 2 min followed by 35 cycles of 95 °C for 30 s, 50 °C for 30 s, and 72 °C for 1 min and ended with an extension step at 72 °C for 5 min. Products of the two reactions from the same sample were pooled and purified with HiYield PCR Clean-up & Gel-Extraction kit (SLG) followed by quantification with Quant-iT PicoGreen dsDNA assay (Invitrogen, Darmstadt, Germany). Equimolar amounts of the purified PCR products were pooled and sent to StarSEQ (Mainz, Germany) for 250 bp paired-end sequencing on a MiSeq instrument. The sequence data was processed with dada2 version 1.6.0^36^ in R 3.4.1 (www.r-project.org). Forward and reverse reads were truncated at positions 235 and 150, respectively, or at any position with a quality score of two. Reads with ambiguous bases or over two expected errors were discarded. Error models were constructed from one million randomly selected reads. The reads were binned into sequence variants (SVs) based on the error models using the pool option. Forward and reverse sequences were merged, and then chimeric sequences were identified with the removeBimeraDenovo function and removed. SVs were classified based on the SILVA reference release 128^37^ accepting only results with at least 50% bootstrap support. SVs classified as chloroplast or mitochondrial sequences were deleted from the dataset. All sequences were deposited at the European Nucleotide Archive under the accession number PRJEB30355.

### Statistical analyses

Plant biomass was compared between the treatment combinations with Tukey-Kramer tests in JMP 13.0.0 (SAS Institute, Cary, NC). The qPCR results were subjected to log_10_ transformation and analyzed in JMP 13.0.0. Copy numbers in the different salinity treatments were compared with Tukey-Kramer tests in case of the phase 1 bare soil samples with and without straw and the phase 2 rhizosphere samples with straw. In case of the phase 2 rhizosphere samples without straw amendment, t-tests were used to compare the control and low salinity treatment. The effect of the straw amendment under the different salinity levels was assessed with t-tests. SV richness was estimated from the sequencing data using objective Bayes procedure based on negative binomial models^38^ implemented in the breakaway R package version 3.0^39^. Simpson diversity was estimated by bootstrapping with 500 iterations using the ‘resample_estimate’ function of the breakaway package. The mean SV richness and Simpson diversity estimates and their standard errors were used in the ‘betta’ function^40^ to test for significant differences between the control and the low or high salinity treatments, or between the samples with and without straw amendment from the same salinity treatment. For preparing ordination plots, SVs that didn’t have at least 0.1% average relative abundance in the samples included in the ordination were removed from the dataset to decrease sparsity. Remaining zeroes were then replaced with the count zero multiplicative method implemented in the zCompositions R package version 1.1.1^41^ and the data was subjected to centered log-ratio (CLR) transformation to remove compositional effects and correct for differences in sequencing depth^42^. Principal component analysis (PCA) was performed in R with the vegan package version 2.4.6^43^. Variation partitioning from the vegan package was employed on the CLR-transformed data matrices used in PCA to assess the proportion of the variation in the data explained by the salinity treatment and the straw amendment. ALDEx2^44^ from the R package version 1.6.0 was used to identify SVs differentially abundant between the control and the low or high salinity treatments, or between the samples with and without straw amendment from the same salinity treatment. Only SVs with at least 0.1% average relative abundance were included in these analyses. In the ALDEx2 results, significance was assessed based on Welch’s t-test with Benjamini and Hochberg’s correction^45^ to maintain a 10% false discovery rate. Figures summarizing the results were prepared in Cytoscape 3.4.0 (www.cytoscape.org).

## Results

### Effect of salinization and straw amendment on soil chemical parameters and plant growth

The low and high salinization treatments during phase 1 increased the electric conductivity in the soil, as intended, but did not alter the pH (Table 1). The straw amended soils had a higher C:N ratio and were slightly more neutral in pH. Theoretically, the straw amendment should have increased the total soil C by 0.96% (w/w) but the difference between the soil with and without straw at the end of phase 1 was lower, possibly due to microbial mineralization during the 11 weeks of incubation. In the bulk soil at the end of phase 2, the EC was slightly lower than in the bare soil at the end of phase 1. Nevertheless, the differences between the control, low, and high salinity treatments were still large, indicating that the salinity stress persisted during the cultivation of wheat.

**Table 1:** Soil chemical parameters (average ± SD) in phase 1 bare soil and in phase 2 bulk soil in the different salinity treatments.

Water treatment	Total soil C (%, w/w)	Total soil N (%, w/w)	C:N ratio	pH	EC_1:5_ [dS m^−1^]
**Phase 1: bare soil after the salinization treatment**					
Soil without straw					
Control	0.28 ± 0.02	0.033 ± 0.005	8.5 ± 1.5	7.8 ± 0.3	0.076 ± 0.004
Low salinity	0.29 ± 0.01	0.037 ± 0.003	8.1 ± 1.0	7.7 ± 0.0	0.348 ± 0.018
High salinity	0.29 ± 0.01	0.035 ± 0.004	8.5 ± 1.1	7.6 ± 0.3	1.728 ± 0.066
Soil amended with straw					
Control	0.98 ± 0.03	0.059 ± 0.005	16.7 ± 1.5	7.5 ± 0.1	0.179 ± 0.003
Low salinity	0.96 ± 0.09	0.056 ± 0.006	17.3 ± 2.5	7.5 ± 0.0	0.465 ± 0.018
High salinity	1.12 ± 0.13	0.054 ± 0.009	21.2 ± 3.9	7.5 ± 0.0	1.678 ± 0.072
**Phase 2: bulk soil after plant growth**					
Soil without straw					
Control	0.31 ± 0.01	0.034 ± 0.005	9.0 ± 0.8	7.6 ± 0.0	0.147 ± 0.032
Low salinity	0.31 ± 0.01	0.029 ± 0.005	10.8 ± 1.8	7.6 ± 0.0	0.280 ± 0.061
High salinity^1^	0.31 ± 0.01	0.025 ± 0.004	12.6 ± 1.9	7.7 ± 0.0	1.612 ± 0.206
Soil amended with straw					
Control	0.83 ± 0.04	0.045 ± 0.006	18.4 ± 1.7	7.3 ± 0.0	0.153 ± 0.006
Low salinity	0.99 ± 0.03	0.043 ± 0.004	23.2 ± 2.0	7.4 ± 0.0	0.347 ± 0.036
High salinity	1.07 ± 0.11	0.047 ± 0.005	22.9 ± 2.3	7.4 ± 0.0	1.153 ± 0.076

The low salinity level stimulated, regardless whether the soil had been amended with straw, the shoot growth of the wheat plants. Under high salinity, however, the seedlings did not survive in the soil without straw. In the straw amended soil the shoot biomass was not significantly different under control and high salinity conditions (Table S3). Root growth was strongly stimulated by the straw amendment under the non-saline control conditions. Low salinity had no significant effect on the root mass but it was significantly reduced under high salinity.

### Abundances of microbial groups based on qPCR

In the bare soil from phase 1, the straw amendment caused a strong increase in bacterial and fungal abundance regardless of the level of salinity (*p* < 0.001) but slightly decreased the abundance of *Archaea* (*p* < 0.005) as indicated by qPCR (Table 2A). Without the straw amendment, there was a small but significant increase in soil bacterial and archaeal abundance in the high salinity treatment compared to the control, but no significant difference was found between the control and the low salinity treatment. In the straw amended soil, the salinity treatment did not affect bacterial abundance, while archaeal rRNA gene numbers were slightly higher under high salinity than in the control. Soil salinity had no significant effect on fungal abundance in the bare soil regardless of the straw amendment.

**Table 2:** 16S rRNA gene or ITS sequence numbers (log_10_ average ± SD) of microbial groups per g soil (dry weight) in bare soil after phase 1 and per g root (fresh weight) in the rhizosphere of wheat from phase 2.

Treatments	Control	Low salinity	High salinity
**A**. **Phase 1: bare soil**			
Soil without straw			
*Bacteria*	9.38 ± 0.04 B	9.42 ± 0.05 AB	9.47 ± 0.02 A
*Archaea*	8.08 ± 0.05 B	8.11 ± 0.05 AB	8.19 ± 0.03 A
*Fungi*	6.91 ± 0.09 A	7.03 ± 0.19 A	6.96 ± 0.04 A
Soil amended with straw			
*Bacteria*	10.30 ± 0.02 A	10.31 ± 0.03 A	10.23 ± 0.02 A
*Archaea*	7.84 ± 0.03 B	7.91 ± 0.05 AB	7.93 ± 0.04 A
*Fungi*	9.13 ± 0.13 A	9.04 ± 0.10 A	9.18 ± 0.10 A
**B**. **Phase 2: rhizosphere soil**.			
Soil without straw			
*Bacteria*	10.42 ± 0.05*	10.09 ± 0.08*	No plant growth
*Archaea*	7.99 ± 0.14	8.08 ± 0.13	
*Fungi*	9.03 ± 0.24	9.63 ± 0.40	
Soil amended with straw			
*Bacteria*	10.44 ± 0.10 A	10.58 ± 0.09 A	10.62 ± 0.10 A
*Archaea*	7.63 ± 0.14 B	7.92 ± 0.20 AB	8.06 ± 0.13 A
*Fungi*	9.44 ± 0.13 A	9.41 ± 0.10 A	9.12 ± 0.28 A

Different capital letters indicate significant (P < 0.05) differences within the same line of the table based on Tukey-Kramer tests. *Indicates significant (P < 0.05) differences based on t-tests.

In the rhizosphere (phase 2), the straw amendment had only minor effects on microbial abundance: Under non-saline conditions it decreased archaeal rRNA gene numbers (*p* = 0.03), and under low salinity it increased bacterial abundance (*p* = 0.002). Soil salinity caused a decrease in bacterial abundance without the straw amendment but had no such effect in the rhizosphere of plants grown in the straw amended soil (Table 2B). *Archaea*, on the other hand, became more abundant under high salinity with the straw amendment compared to the control. Fungal abundance in the rhizosphere was neither affected by the straw amendment nor the salinization.

### Microbial richness and diversity in the bare soil and wheat rhizosphere

**Figure 1:**
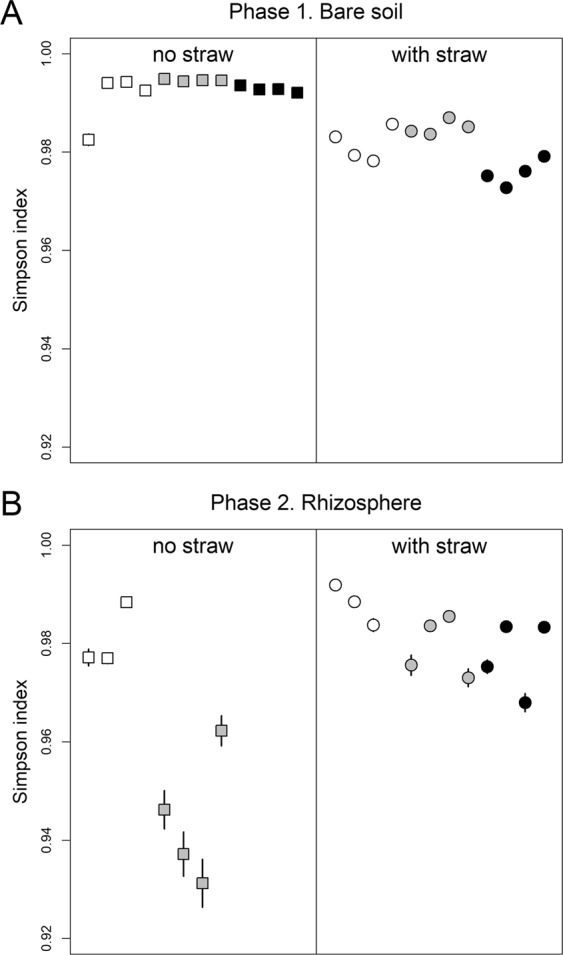
Estimated Simpson diversity index of the bacterial and archaeal community in (**A**) the bare soil from phase 1 and (**B**) in the rhizosphere from phase 2. Circles and squares indicate the mean estimates for each sample, lines the standard errors. Control: empty symbols; low salinity: grey symbols; high salinity: black symbols.

From the bare soil samples (phase 1), 1,729,506 high-quality sequences (47,219 to 95,177 sequences per sample) were obtained by sequencing of the prokaryotic 16S rRNA gene amplicons. In each sample 96.9 to 99.8% of the sequences were assigned to the following ten phyla: *Firmicutes* (23.4–46.7%), *Proteobacteria* (13.3–43.7%), *Actinobacteria* (8.0–38.1%), *Thaumarchaeota* (0.2–7.1%), *Gemmatimonadetes* (0.5–5.6%), *Chloroflexi* (0.7–4.1%), *Bacteroidetes* (0.3–3.7%), *Acidobacteria* (0.1–3.3%), *Planctomycetes* (0.3–2.2%), and *Verrucomicrobia* (0.0–4.0%) (Table S4). The five most abundant classes were *Bacilli* (23.2 to 46.7%), *Alphaproteobacteria* (4.9 to 35.9%), *Gammaproteobacteria* (1.4 to 6.7%), *Thermoleophilia* (1.3 to 20.3%), and *Actinobacteria* (5.0 to 16.3%). The straw amendment significantly decreased the richness and the diversity of the bacterial and archaeal community in the bare soil (P < 0.001) (Figs 1A and S1A). High salinity had a negative effect on richness (*p* < 0.05) which was stronger with the straw amendment than without it. The salinity treatment, however, did not have a significant effect on diversity according to the Simpson index.

From the rhizosphere samples (phase 2), a total of 1,010,317 high-quality sequences (21,521 to 93,126 sequences per sample) were retrieved. In each sample 97.5 to 99.6% of the sequences were from the ten most dominant phyla: *Proteobacteria* (36.8–56.2%), *Firmicutes* (1.3–30.0%), *Actinobacteria* (7.0–20.3%), *Verrucomicrobia* (1.9–27.6%), *Bacteroidetes* (2.7–14.7%), *Gemmatimonadetes* (0.7–12.7%), *Chloroflexi* (0.4–4.8%), *Planctomycetes* (1.1–3.5%), *Acidobacteria* (0.0–3.9%), and *Thaumarchaeota* (0.1–3.8%) (Table S4). The five most abundant classes in the rhizosphere were *Alphaproteobacteria* (19.1 to 43.8%), *Gammaproteobacteria* (4.1 to 25.7%), *Bacilli* (1.2 to 26.7%), *Verrucomicrobiae* (0.2 to 25.2%), and *Actinobacteria* (4.5 to 16.3%). The straw amendment significantly decreased (*p* < 0.001) the richness of the bacterial and archaeal community in the rhizosphere (Fig. S1B). However, it mitigated the negative effect of salinity on Simpson diversity: both the low and the high salinity treatments had a significant negative effect on diversity (*p* < 0.004) but it was stronger in the rhizosphere of plants grown in the soil without straw added (Fig. 1B).

### Responses of the microbial community structure to salinity and straw amendment

**Figure 2:**
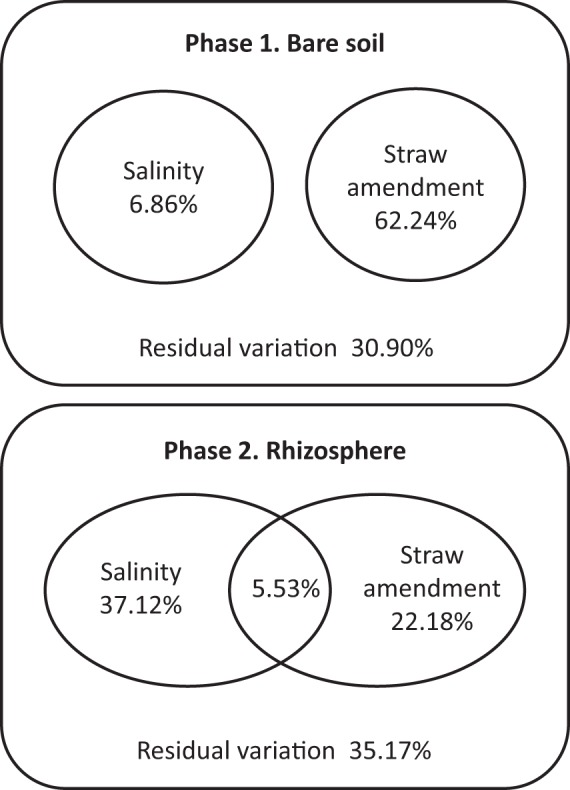
Variation partitioning results. The percentages are the proportion of the variation in the sequencing results which could be explained by salinity or straw amendment. The shared partitions indicate the proportion of variation explained by both factors.

**Figure 3:**
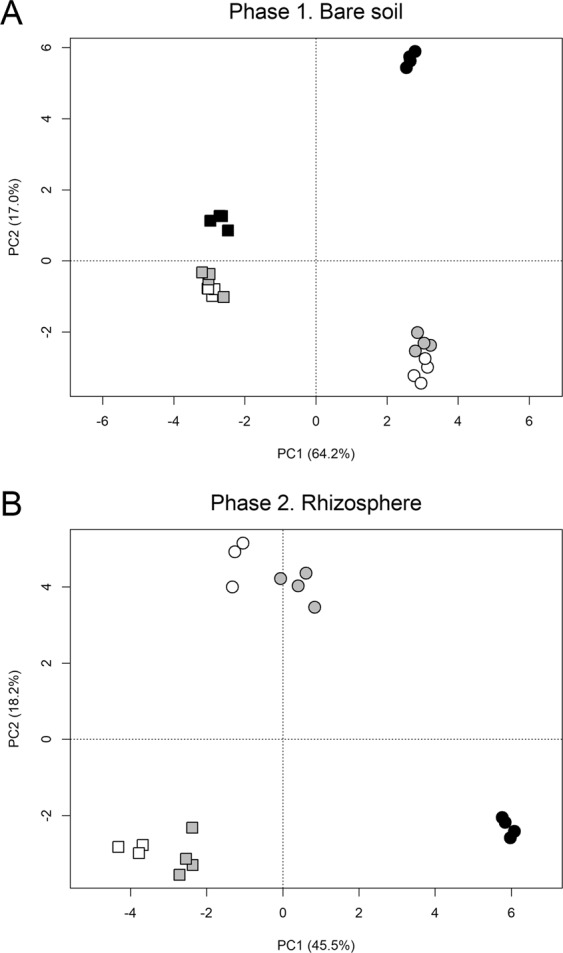
Principal component analysis plots of phase 1 bare soil (**A**) and phase 2 wheat rhizosphere samples (**B**). Circles indicate samples from the straw amendment, squares indicate samples without straw. Control: empty symbols; low salinity: grey symbols; high salinity: black symbols.

The effects of soil salinization and straw amendment on the structure of the bacterial and archaeal communities were quantified and compared with variation partitioning and the differences in community structure between the samples were visualized by PCA. Among the bare soil samples from phase 1, the straw amendment had a large effect on the community structure explaining the majority of the variation in the data (Fig. 2A). Comparably, the effect of soil salinization was 9-fold smaller. The control and the low salinity samples don’t separate strongly on the PCA plot (Fig. 3A). The effect of the high salinity treatment was larger and especially pronounced in the straw amended soil.

In the rhizosphere samples from phase 2, salinity had a larger influence on the bacterial and archaeal community structure, explaining 42.7% of the variation in the data, than the straw amendment which could account for 27.7% (Fig. 2B). In contrast to the bare soil, soil salinization already at a low level caused a change in community structure in the rhizosphere as indicated by the clear separation between the control and low salinity samples on the PCA plot. The high-level salinity, which could only be tested in straw-amended soil, had an even stronger influence on the community structure (Fig. 3B).

### SVs responding to soil salinization

**Figure 4:**
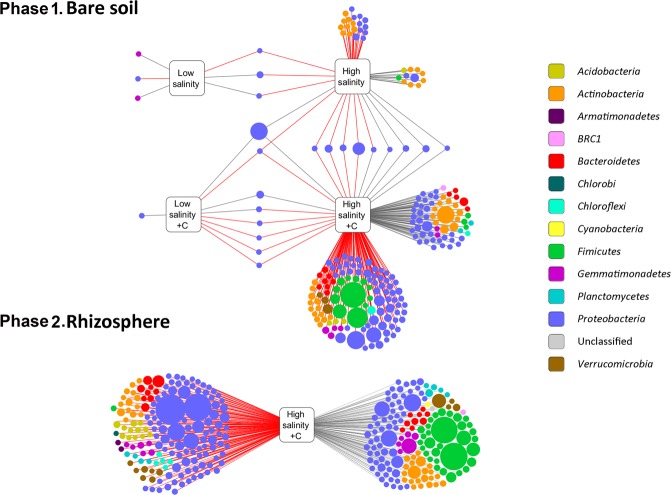
SVs responding to soil salinity. Circles represent SVs colored according to their phylum-level classification and sized by their average relative abundance. Lines connect SVs to treatments in which their abundance changed significantly compared to the control with the corresponding carbon treatment. Black lines indicate significant increase, red lines significant decrease.

In bare soil from phase 1 without the straw amendment, only six SVs showed a significant change in relative abundance in response to the low salinity treatment: Four SVs, two classified as *Proteobacteria* and two as *Gemmatimonadetes*, increased while two from *Proteobacteria* decreased (Fig. 4, upper panel). In contrast, a total of 44 SVs responded to the high salinity treatment (16 increased, 28 decreased). Most of them were classified as *Actinobacteria* and *Proteobacteria*. There was no apparent link between the taxonomic classification of these SVs and the direction of their response to salinity. All SVs that were found to be significantly differentially abundant between treatments are listed in supplementary file 1.

In straw amended bare soil, nine SVs, all of them belonging to *Proteobacteria*, responded significantly to the low salinity treatment while in the high salinity treatment the relative abundance of 67 SVs increased and 115 SVs decreased significantly compared to the control (Fig. 4, upper panel). The 67 SVs represented eight phyla, the most dominants being *Proteobacteria* and *Actinobacteria*. The 115 SVs that responded negatively to high salinity were members of nine phyla including *Proteobacteria*, *Actinobacteria* and *Firmicutes*. The most abundant among these were two SVs from family *Bacillaceae* (*Firmicutes*) with 2.24% and 1.54% average relative abundance.

In the phase 2 rhizosphere samples, no SV with a relative abundance larger than 0.1% was found to have a significant response to the low salinity treatment, regardless whether the soil was previously amended with straw or not. However, in the straw-amended soils, 299 SVs showed a significant response to the high salinity treatment: 144 SVs increased and 155 SVs decreased in relative abundance (Fig. 4, lower panel). These SVs covered a wide range of taxonomic diversity representing 13 phyla with a large overlap in the classification of the SVs that responded positively and the ones that responded negatively. A strong difference, however, was found among *Firmicutes* which, except for a single SV, were only present among the SVs that increased in relative abundance under high salinity. The two most dominants of these SVs were from *Planococcaceae* with 2.24% and 2.17% average relative abundances. The others belonged to *Planococcaceae*, *Bacillaceae*, and *Paenibacillaceae*. In contrast, *Acidobacteria* were only present among the SVs that decreased in relative abundance under high salinity with 12 SVs, most of them classified into *Blastocatellaceae* and subgroups 6 and 10.

### SVs responding to the straw amendment

**Figure 5:**
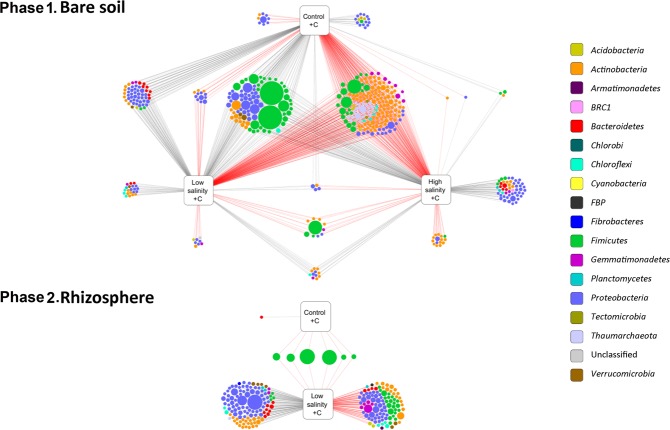
SVs responding to the straw amendment. Circles represent SVs colored according to their phylum-level classification and sized by their average relative abundance. Lines connect SVs to treatments in which their abundance changed significantly compared to the corresponding salinity treatment without the carbon amendment. Black lines indicate significant increase, red lines significant decrease.

In the bare soil from phase 1, the number of SVs that significantly responded to the straw amendment (468 SVs) was much higher than the number of SVs affected by salinity (219 SVs). The majority, 251 out of 468, of the SVs that changed in relative abundance in response to the straw amendment did so independent of the level of salinity: 79 of them increased while 172 decreased (Fig. 5, upper panel). Most of the 79 SVs that showed significant increase were classified as *Bacillales* (*Firmicutes*), *Alphaproteobacteria* (*Proteobacteria*), and *Actinobacteria*. The most dominants of them were two SVs from *Planococcaceae* (*Firmicutes*) with 3.93% and 3.90% relative abundances. In comparison, the 172 SVs that responded negatively to the straw amendment under all salinity conditions encompassed a larger taxonomic diversity with members of eight phyla. *Actinobacteria* was dominant in this group with numerous SVs classified as *Rubrobacter*, *Solirubrobacter*, and *Gaiellales*. Other taxa represented by several SVs in this group included *Thaumarchaeota*, *Deltaproteobacteria*, *Gemmatimonadetes*, and *Bacillaceae* and *Paenibacillaceae* (*Firmicutes*). Only a small number of SVs showed a significant response to the straw amendment in just the control (26 SVs) or just the low salinity (29 SVs) treatments. In contrast, 19.5% of the SVs that significantly changed their relative abundance under high salinity due to the straw amendment were not found to respond significantly in the control and low salinity treatments. Most of these SVs were classified as *Alphaproteobacteria* and *Actinobacteria*.

In the rhizosphere samples from phase 2, only seven SVs were found to respond significantly to the straw amendment under the non-saline control conditions (Fig. 5, lower panel). Except for one, these SVs also increased in relative abundance due to the straw addition under low salinity. They were classified as *Bacillales* (*Firmicutes*) and included the two abundant *Planococcaceae* SVs that also responded to the straw amendment, independent of the level of salinity, in the bare soil in phase 1. In contrast to the non-saline control conditions, 217 SVs reacted to the straw amendment in the rhizosphere under low salinity, 106 of them with an increase and 111 with a decrease in relative abundance. *Firmicutes*, *Proteobacteria*, and *Actinobacteria* were dominant among the SVs that increased, and *Proteobacteria* and *Actinobacteria* among the ones that showed a negative response.

## Discussion

The experimental design of this study allowed distinguishing the effect of a low-level of soil salinization which was stimulatory to the shoot growth of wheat plants, from a high-level salinity which inhibited plant growth unless straw was added to the soil. This demonstrates the efficiency of straw amendment in ameliorating salinity stress on wheat. Under non-saline conditions, root growth was strongly stimulated by the straw amendment likely due to the additional nitrogen and other minerals provided by the straw. Furthermore, the 1 mm-sized straw particles could have altered the soil structure thereby facilitating root growth. Additions of organic particles to soils have been shown to affect root morphology and biomass^46^. The straw amendment could promote plant growth by counteracting soil compaction which is an important factor in the adverse effects on wheat in salt affected soils^47^.

The soil used in this study had not experienced previous treatments with saline water, and we hypothesized that the addition of saline water to the bare soil would therefore mobilize soil organic carbon and other nutrients^19,48^ which would support microbial growth. Such growth would result in higher microbial abundance but concomitantly decrease microbial diversity due to enrichment of a few community members. Salinization did increase the abundance of *Bacteria* and *Archaea* in bare soil. However, without straw amendments this effect was found to be small and only detectable under high-level salinity. Fungal abundance was unaffected by soil salinization. Considering the relatively low C content of the soil used in this study, the quantity of this mobilized carbon may have been too small to support strong growth, or this effect was only transient and mostly dissipated over the three weeks equilibration period between the end of the salinization treatment and the sampling. The lack of a strong response in diversity or community structure to salinization in the bare soil was therefore presumably due to the low level of microbial activity. Accordingly, providing available carbon in the form of the straw amendment enhanced the effect of salinity on the richness and structure of the bacterial and archaeal community, largely increasing the number of SVs that changed in relative abundance in response to high-level salinity. Based on the qPCR data, *Bacteria* and especially *Fungi* were the main beneficiaries of the straw additions, but the abundance of *Archaea* significantly declined, indicating that they played a negligible role in straw decomposition. In accordance, the relative abundance of SVs affiliated to *Thaumarchaeota* decreased in relative abundance in response to straw additions. Most of these archaeal SV were affiliated with *Nitrososphaera*, which are suspected to perform autotrophic nitrification in soil^49^. Thus, they are probably less competitive when organic carbon, as supplied by straw, becomes available. SVs representing *Gemmatimonadetes* also exclusively declined in response to the straw amendment. The addition of wheat straw to soil reduced *Gemmatimonadetes* in other studies as well^50^.

In the rhizosphere, our hypothesis that salinity would decrease microbial diversity was found to be true even under the low-level salinity treatment. Salinity also had a much stronger influence on the bacterial and archaeal community structure in the rhizosphere than in the bare soil. As opposed to the bare soil, the rhizosphere is a hot spot of microbial activity where carbon is available from plant root exudates^51,52^. In this sense, it is more similar to the bare soil with straw amendment where the effect of salinization was also more pronounced. Additionally, physiological changes induced by the salinity stress in the plant^13,14^ can contribute to the microbial responses in the rhizosphere.

A striking difference was found in the response of *Firmicutes* SVs to the high-level salinization between bare soil and rhizosphere. In bare soil, they were dominant among the SVs that declined in relative abundance, while in the rhizosphere numerous *Firmicutes* SVs increased. The phylum *Firmicutes* thus contains taxa with contrasting survival capacities: one group of SVs represented mainly by *Tumebacillus* and *Bacillaceae* that appeared to grow on the nutrients supplied by straw but were sensitive to salinity, while other SVs, mainly members of *Planococcaceae* but also *Paenibacillaceae* and *Bacillaceae*, were abundant in the rhizosphere and tolerant to salinity. All cultivable members of the *Planococcaceae* isolated from a wide range of relatively extreme environments but not from rhizosphere and semiarid soil, are halotolerant^53^. Salt-tolerance is also a relatively common property of many bacilli. The fact that members of *Bacillaceae* occurred in both groups (bare soil – salt-sensitive; rhizosphere – salt-resistant) underlines the high diversity of this taxon, which becomes evident when considering their wide ecological range^54,55^.

Interestingly, the straw amendment enhanced the effect of soil salinization on bacterial and archaeal richness and community structure in the bare soil, but it mitigated the salinity effect on diversity in the rhizosphere. The former can be explained by the nutrients delivered by the straw inducing microbial growth in the bare soil rendering the community more susceptible to the selective pressure of salinity compared to the less active community in the soil without the straw amendment. In the rhizosphere, on the other hand, carbon is available from root exudates and other rhizodeposits^56^, and, as shown by the qPCR results, the nutrients from the straw amendment had only minor impact on microbial growth. Therefore, the straw likely decreased the effect of salinity on the rhizosphere microbiota indirectly, by mitigating the salinity stress on the plant.

The results of this study demonstrate the importance of soil microhabitats with different conditions, i.e. the rhizosphere or the presence of straw particles, in modulating the response of soil microbial communities to salinization in semi-arid soils. Contrasting responses of the microbial community were detected. Soil salinization had little or no detectable effect on microbial abundance and prokaryotic diversity in the soil when no external carbon source was provided. On the other hand, it decreased prokaryotic diversity in the rhizosphere of wheat grown in the soil. Straw amendment mitigated this effect. However, it was not a significant nutrient source for the microbial communities in the rhizosphere but more likely acted indirectly by ameliorating the salinity stress on the plant. Diverse responses were found within *Proteobacteria*, *Actinobacteria*, and *Firmicutes* to salinity and the straw amendment indicating the large physiological versatility within these highly diversified phyla.

## Supplementary information

Supplementary information

Supplementary file 1

## Data Availability

Sequencing data generated in this study is available from the European Nucleotide Archive under the accession number PRJEB30355.
