# Supplementary material for: Contrasting microbial community responses to salinization and straw amendment in a semiarid bare soil and its wheat rhizosphere
Source: Sci Rep. 2019 Jul 5;9:9795. doi: 10.1038/s41598-019-46070-6 (PMC6611862; doi:10.1038/s41598-019-46070-6)
Supplement: Supplementary file 1 — Supplementary information [file 41598_2019_46070_MOESM1_ESM.pdf]

Supplementary information for

Contrasting microbial community responses to salinization and straw amendment in a semiarid bare soil and its wheat rhizosphere

Márton Szoboszlay[1]*, Astrid Näther[1]*, Bei Liu[1,2], Angel Carrillo[3], Thelma Castellanos[3], Kornelia Smalla[4], Zhongjun Jia[2], Christoph C. Tebbe[1]**

[1]Thünen Institute of Biodiversity, Braunschweig, Germany; [2]Institute of Soil Science, Chinese Academy of Science, Nanjing, Jiangsu, China; [3]Centro de Investigaciónes Biologicas del Noroeste (CIBNOR), La Paz, Baja California Sur, Mexico; [4]Julius Kühn Institute of Epidemiology and Pathogen Diagnostics, Braunschweig, Germany

*, both authors contributed equally to this manuscript

**, corresponding author

Thünen Institut für Biodiversität, Bundesallee 65, 38116 Braunschweig

E-mail: christoph.tebbe@thuenen.de

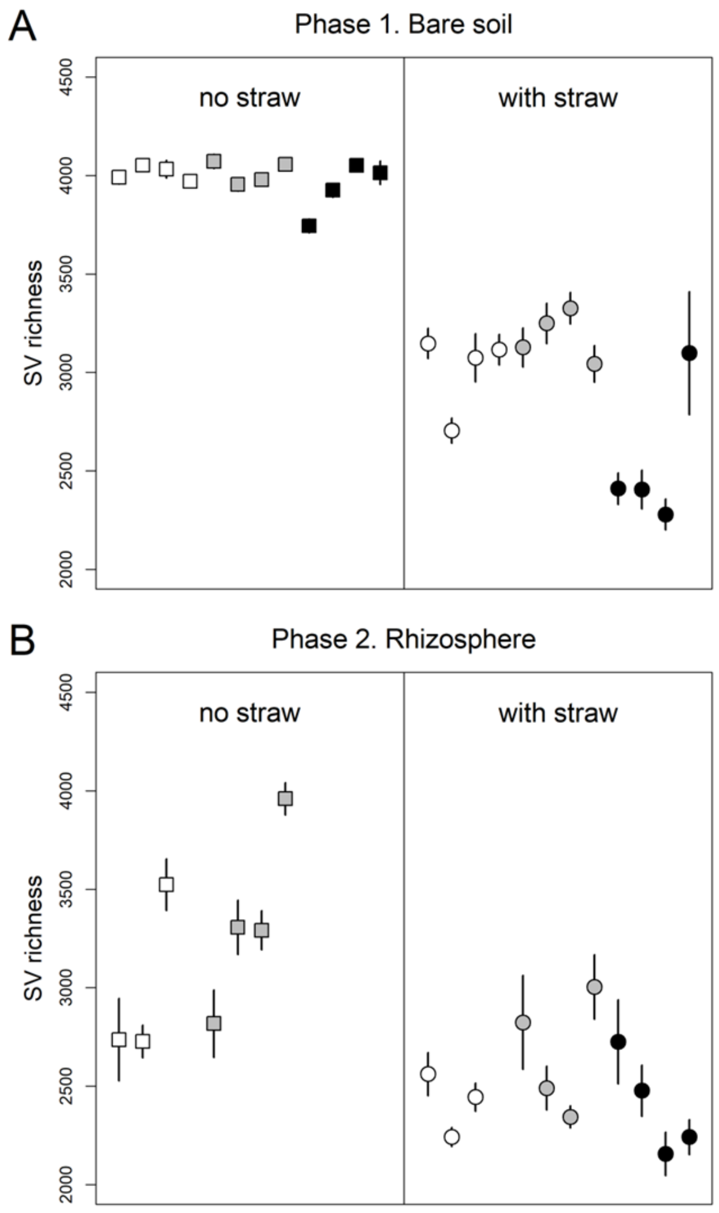

Figure S1: Estimated richness of the bacterial and archaeal community in (A) the bare soil from phase 1 and (B) in the rhizosphere from phase 2. Circles and squares indicate the mean estimates for each sample, lines the standard errors. Control: empty symbols; low salinity: grey symbols; high salinity: black symbols.

Table S1: Primers, probes, and PCR conditions for the quantification of *Bacteria*, *Archaea*, and *Fungi* by qPCR

	Primer/Probe	Thermal profile	Reference
**Bacteria**<br>16S rRNA<br>468 bp	BAC338F (5'-ACTCCTACGGGAGGCAG-3')<br>BAC516F (5'-TGCCAGCAGCCGCGGTAATAC-3')<br>BAC805R (5'-GACTACCAGGGTATCTAATCC-3')	95 °C – 10 min<br>40 x (95 °C: 15 s, 60 °C: 60 s)	1
**Archaea**<br>16S rRNA<br>273 bp	ARC787F (5'- ATTAGATACCCSBGTAGTCC-3')<br>ARC915F (5'- AGGAATTGGCGGGGGAGCAC-3')<br>ARC1059R (5'-GCCATGCACCWCCTCT-3')	95 °C – 10 min<br>40 x (95 °C: 15 s, 60 °C: 60 s)	1
**Fungi**<br>ITS<br>300-500 bp	NSI1 (5'- GATTGAATGGCTTAGTGAGG-3')<br>58A2R (5'- CTGCGTTCTTCATCGAT-3')	95 °C – 10 min<br>40 x (9 5°C: 15 s, 52 °C: 30 s, 72 °C: 30 s)	2

1. Yu, Y., Lee, C., Kim, J. & Hwang, S. Group-specific primer and probe sets to detect methanogenic communities using quantitative real-time polymerase chain reaction. Biotechnol Bioeng 89, 670-679 (2005).
2. Martin, K. J. & Rygiewicz, P. T. Fungal-specific PCR primers developed for analysis of the ITS region of environmental DNA extracts. BMC Microbiology 5, 28, doi:10.1186/1471-2180-5-28 (2005).

Table S2: Primers used in the amplification of 16S rRNA gene sequences for Illumina sequencing

**Forward Illumina adapter**	**Forward**	**Forward pad**	**Forward**	**Forward primer: S-D-Arch-**
AATGATACGGCGACCACCGAGATCTACAC	ACTATCTG	GATGGTAATC	AT	CAGCMGCCGCGGTAA
AATGATACGGCGACCACCGAGATCTACAC	TAGCGAGT	GATGGTAATC	AT	CAGCMGCCGCGGTAA
AATGATACGGCGACCACCGAGATCTACAC	CGATCTAC	GATGGTAATC	AT	CAGCMGCCGCGGTAA
AATGATACGGCGACCACCGAGATCTACAC	GTCGAGCA	GATGGTAATC	AT	CAGCMGCCGCGGTAA
AATGATACGGCGACCACCGAGATCTACAC	GATCGTGT	GATGGTAATC	AT	CAGCMGCCGCGGTAA
AATGATACGGCGACCACCGAGATCTACAC	ATATACAC	GATGGTAATC	AT	CAGCMGCCGCGGTAA
AATGATACGGCGACCACCGAGATCTACAC	TGCGTACG	GATGGTAATC	AT	CAGCMGCCGCGGTAA
AATGATACGGCGACCACCGAGATCTACAC	CCGACGTA	GATGGTAATC	AT	CAGCMGCCGCGGTAA
AATGATACGGCGACCACCGAGATCTACAC	GGTAGCGT	GATGGTAATC	AT	CAGCMGCCGCGGTAA
AATGATACGGCGACCACCGAGATCTACAC	CTACTATA	GATGGTAATC	AT	CAGCMGCCGCGGTAA
AATGATACGGCGACCACCGAGATCTACAC	TACGAGAC	GATGGTAATC	AT	CAGCMGCCGCGGTAA
AATGATACGGCGACCACCGAGATCTACAC	ACGTCTCG	GATGGTAATC	AT	CAGCMGCCGCGGTAA
**Reverse Illumina adapter**	**Reverse**	**Reverse pad**	**Reverse**	**Reverse primer: S-D-Bact-**
CAAGCAGAAGACGGCATACGAGAT	AACTCTCG	AGTCAGTCAG	GA	GACTACHVGGGTATCTAATCC
CAAGCAGAAGACGGCATACGAGAT	CGTAGATC	AGTCAGTCAG	GA	GACTACHVGGGTATCTAATCC
CAAGCAGAAGACGGCATACGAGAT	GCGCACGT	AGTCAGTCAG	GA	GACTACHVGGGTATCTAATCC
CAAGCAGAAGACGGCATACGAGAT	TTAGTGAA	AGTCAGTCAG	GA	GACTACHVGGGTATCTAATCC

Table S3: Root and shoot biomass (average ± SD dry weight, d.w.) of the wheat plants at sampling. Capital letters indicate significant differences in root mass or shoot mass according to Tukey-Kramer tests

	Root mass (g d.w.)		Shoot mass (g d.w.)	
	No straw	With straw	No straw	With straw
Control	0.08 ± 0.01 B	0.40 ± 0.14 A	0.15 ± 0.03 B	0.14 ± 0.05 B
Low salinity	0.13 ± 0.09 B	0.25 ± 0.10 AB	0.27 ± 0.01 A	0.27 ± 0.08 A
High salinity	No growth	0.10 ± 0.07 B	No growth	0.19 ± 0.02 AB

Table S4: Relative abundances of the dominant prokaryotic phyla and classes in the phase 1 bare soil and phase 2 rhizosphere samples expressed as percentages (average ± SD)

**Soil type**			**Bare soil**						**Wheat rhizosphere**				
Straw amendment			Soil without straw			Soil amended with straw			Soil without straw		Soil amended with straw		
Salinity treatment			Control	Low salinity	High salinity	Control	Low salinity	High salinity	Control	Low salinity	Control	Low salinity	High salinity
Number of sequences			66,138 ± 9,567	63,523 ± 4,755	76,313 ± 17,707	82,602 ± 6,704	80,309 ± 6,321	63,313 ± 10,926	54,269 ± 30,101	41,991 ± 14,815	42,729 ± 16,494	47,471 ± 24,369	90,368 ± 5,031
**Domain**	**Phylum**	**Class**											
Archaea			5.44 ± 1.14	5.96 ± 0.33	4.90 ± 0.82	0.25 ± 0.05	0.31 ± 0.08	0.29 ± 0.03	1.36 ± 2.09	0.32 ±0.20	0.15 ± 0.10	0.13 ± 0.08	0.16 ± 0.02
	Thaumarchaeota		5.42 ± 1.13	5.94 ± 0.32	4.90 ± 0.81	0.25 ± 0.05	0.31 ± 0.08	0.29 ± 0.03	1.35 ± 2.10	0.32 ± 0.20	0.15 ± 0.10	0.13 ± 0.08	0.16 ± 0.02
		Soil Crenarchaeotic Group	5.42 ± 1.13	5.94 ± 0.32	4.90 ± 0.81	0.25 ± 0.05	0.31 ± 0.08	0.29 ± 0.03	1.35 ± 2.10	0.32 ± 0.20	0.15 ± 0.10	0.13 ± 0.08	0.16 ± 0.02
Bacteria			94.6 ± 1.1	94.0 ± 0.3	95.1 ± 0.8	99.8 ± 0.1	99.7 ± 0.1	99.7 ± 0.0	98.6 ± 2.1	99.7 ± 0.2	99.9 ± 0.1	99.9 ± 0.1	99.8 ± 0.0
	Firmicutes		24.2 ± 1.3	25.0 ± 0.3	29.7 ± 1.4	40.4 ± 4.5	36.7 ± 2.0	43.8 ± 2.8	3.30 ± 2.56	4.56 ± 1.57	18.0 ± 3.6	19.3 ± 1.9	25.9 ± 3.2
		Bacilli	23.9 ± 1.3	24.7 ± 0.3	29.8 ± 1.5	40.4 ± 4.5	36.6 ± 2.0	43.8 ± 2.8	3.20 ± 2.46	4.43 ± 1.59	17.8 ± 3.6	19.2 ± 19	24.0 ± 2.0
	Proteobacteria		24.3 ± 5.8	18.0 ± 1.0	16.0 ± 2.2	39.9 ± 2.8	38.2 ± 3.3	33.8 ± 1.5	51.5 ± 4.1	46.8 ± 4.2	47.7 ± 3.0	42.5 ± 3.9	43.9 ± 2.1
		Alphaproteobacteria	6.42 ± 1.01	8.45 ± 0.41	6.30 ± 1.75	32.2 ± 2.7	29.9 ± 2.4	30.0 ± 1.7	32.6 ± 11.6	34.6 ± 2.0	32.6 ± 4.0	31.9 ± 1.6	23.1 ± 4.0
		Betaproteobacteria	11.5 ± 7.5	4.02 ± 0.81	1.60 ± 1.25	1.35 ± 0.18	1.28 ± 0.13	0.24 ± 0.07	5.30 ± 1.41	1.86 ± 0.23	2.60 ± 1.60	1.28 ± 0.54	0.27 ± 0.16
		Deltaproteobacteria	4.68 ± 0.64	3.70 ± 0.19	2.49 ± 0.29	1.51 ± 0.20	1.78 ± 0.25	0.71 ±0.24	2.76 ± 1.58	4.26 ± 1.04	4.55 ± 0.81	2.26 ± 0.44	1.24 ± 0.55
		Gammaproteobacteria	1.67 ± 0.20	1.82 ± 0.19	5.51 ± 0.63	4.83 ± 0.41	5.31 ± 1.04	2.83 ± 0.48	10.7 ± 9.1	6.14 ± 1.98	7.91 ± 2.28	7.01 ± 1.92	19.3 ± 4.9
	Actinobacteria		31.6 ± 2.6	34.2 ± 0.5	36.4 ± 1.8	10.9 ± 2.3	14.4 ± 2.8	16.0 ± 2.0	17.4 ± 3.3	11.8 ± 3.6	9.53 ± 4.12	11.5 ± 1.0	12.2 ± 3.7
		Actinobacteria	5.77 ± 0.77	6.21 ± 0.08	8.13 ± 0.07	8.62 ± 1.66	11.4 ± 2.6	13.7 ± 1.9	12.2 ± 3.3	5.90 ± 1.14	6.61 ± 2.51	9.24 ± 1.01	11.1 ± 3.6
		Rubrobacteria	3.64 ± 0.79	4.64 ± 0.69	5.50 ± 0.28	0.16 ± 0.05	0.22 ± 0.05	0.24 ± 0.04	0.29 ± 0.13	0.86 ± 0.47	0.08 ± 0.01	0.13 ± 0.03	0.09 ± 0.01
		Thermoleophilia	18.7 ± 1.0	19.4 ± 0.4	18.8 ± 1.5	1.84 ± 0.62	2.28 ± 0.39	1.66 ± 0.11	3.71 ± 3.09	3.97 ± 1.75	2.28 ± 1.33	1.74 ± 0.51	0.59 ± 0.11
	Gemmatimonadetes		3.34 ± 0.51	5.19 ± 0.30	3.94 ± 0.28	0.79 ± 0.31	1.33 ± 0.22	0.87 ± 0.38	1.07 ± 0.21	1.45 ± 0.82	1.68 ± 0.43	2.41 ± 0.27	6.48 ± 4.30
		Gemmatimonadetes	2.83 ± 0.44	3.69 ± 0.20	2.84 ± 0.20	0.61 ± 0.25	1.08 ± 0.18	0.31 ± 0.07	0.63 ± 0.23	0.83 ± 0.47	0.54 ± 0.13	0.76 ± 0.31	0.12 ± 0.03
	Chloroflexi		3.34 ± 0.63	3.56 ± 0.21	2.88 ± 0.19	1.51 ± 0.47	2.10 ±0.47	0.82 ± 0.19	3.98 ± 0.75	2.13 ± 0.20	2.66 ± 1.47	2.26 ± 0.49	0.58 ± 0.15
		Thermomicrobia	0.73 ± 0.18	0.75 ± 0.07	0.75 ±0.04	1.29 ± 0.41	1.81 ± 0.39	0.68 ± 0.17	0.61 ± 0.23	0.38 ± 0.08	1.29 ± 0.90	1.46 ± 0.50	0.47 ± 0.12
	Bacteroidetes		0.73 ± 0.09	0.51 ± 0.03	0.95 ± 1.15	1.75 ± 0.38	2.31 ± 0.25	3.13 ± 0.61	5.40 ± 1.34	5.19 ± 0.80	3.63 ± 0.56	6.70 ± 5.37	4.25 ± 1.71
		Sphingobacteriia	0.41 ± 0.05	0.27 ± 0.03	0.10 ± 0.06	1.47 ± 0.23	1.71 ± 0.19	2.53 ± 0.62	4.61 ± 1.15	3.27 ± 0.60	2.43 ± 0.43	4.55 ± 5.84	1.70 ± 0.56
	Acidobacteria		2.29 ± 0.50	3.20 ± 0.17	1.81 ± 0.22	0.79 ± 0.21	0.58 ± 0.12	0.07 ± 0.02	3.16 ± 0.74	1.14 ± 0.19	3.38 ± 0.45	0.95 ± 0.28	0.04 ± 0.01
		Subgroup 6	1.29 ± 0.26	1.70 ± 0.09	1.00 ± 0.16	0.52 ± 0.11	0.35 ± 0.09	0.03 ± 0.01	0.68 ± 0.58	0.49 ±0.11	1.19 ± 0.16	0.38 ± 0.20	0.03 ± 0.01
		Blastocatellia	0.52 ± 0.15	0.64 ± 0.03	0.20 ± 0.01	0.19 ± 0.08	0.17 ± 0.03	0.02 ± 0.01	2.12 ± 0.15	0.38 ± 0.05	1.47 ± 0.30	0.49 ± 0.13	0.01 ± 0.00
	Planctomycetes		1.95 ± 0.22	1.42 ± 0.15	0.73 ± 0.24	1.18 ± 0.28	1.06 ± 0.21	0.47 ± 0.15	1.45 ± 0.32	1.35 ± 0.15	2.68 ± 0.37	1.52 ± 0.20	2.54 ± 0.68
		Planctomycetacia	1.79 ± 0.20	1.30 ± 0.14	0.67 ± 0.21	0.81 ± 0.21	0.72 ± 0.18	0.29 ± 0.06	1.09 ± 0.21	1.16 ± 0.13	2.28 ± 0.45	0.93 ± 0.19	0.67 ± 0.16
	Verrucomicrobia		0.10 ± 0.03	0.11 ± 0.01	0.06 ± 0.04	2.20 ± 1.30	2.69 ± 0.82	0.20 ± 0.03	9.87 ± 4.68	23.9 ± 3.5	9.45 ± 3.39	11.8 ± 2.3	3.32 ± 1.17
		Verrucomicrobiae	0.01 ± 0.00	0.01 ± 0.01	0.01 ± 0.02	1.45 ± 1.15	1.91 ± 0.67	0.00 ± 0.00	7.72 ± 4.20	22.0 ± 3.1	6.50 ± 3.35	9.59 ± 2.46	0.50 ± 0.37
